# Effectiveness of Washing Procedures in Reducing *Salmonella enterica* and *Listeria monocytogenes* on a Raw Leafy Green Vegetable (*Eruca vesicaria*)

**DOI:** 10.3389/fmicb.2016.01663

**Published:** 2016-10-20

**Authors:** Alessandra Pezzuto, Simone Belluco, Carmen Losasso, Ilaria Patuzzi, Paola Bordin, Alessia Piovesana, Damiano Comin, Renzo Mioni, Antonia Ricci

**Affiliations:** ^1^Optimization and Control of Food Production Laboratory, Istituto Zooprofilattico Sperimentale delle VenezieSan Donà di Piave, Italy; ^2^Department of Food Safety, Istituto Zooprofilattico Sperimentale delle VenezieLegnaro, Italy; ^3^Department of Animal Medicine, Production and Health, Università di PadovaPadova, Italy; ^4^Department of Information Engineering, Università di PadovaPadova, Italy

**Keywords:** consumer phase, food safety, fresh produce, microbiological risk, *Salmonella*, *Listeria*

## Abstract

Vegetables are an important source of nutrients, but they can host a large microbial population, particularly bacteria. Foodborne pathogens can contaminate raw vegetables at any stage of their production process with a potential for human infection. Appropriate washing can mitigate the risk of foodborne illness consequent to vegetable consumption by reducing pathogen levels, but few data are available to assess the efficacy of different practices. In the present work, six different washing methods, in the presence or absence of sanitisers (peracetic acid and percitric acid, sodium bicarbonate, sodium hypochlorite) and vinegar, were tested for their effectiveness in reducing *Salmonella* and *Listeria* counts after artificial contamination of raw rocket (*Eruca vesicaria*). Results showed that washing with sodium hypochlorite (200 mg/L) was the only method able to produce a significant 2 Log reduction of *Salmonella* counts, but only in the case of high initial contamination (7 Log CFU/g), suggesting potential harmful effects for consumers could occur. In the case of *Listeria monocytogenes*, all the examined washing methods were effective, with 200 mg/L sodium hypochlorite solution and a solution of peracetic and percitric acids displaying the best performances (2 and 1.5 Log reductions, respectively). This highlights the importance of targeting consumers on fit for purpose and safe washing practices to circumvent vegetable contamination by foodborne pathogens.

## Introduction

Vegetables are an important source of dietary fiber, vitamins and minerals, have low energy density and provide a range of nutrients that are required to regulate the body’s metabolic functions. For these reasons, dietary guidelines recommend a high intake of vegetables (WHO/FAO, 2003).

However, due to their high surface/weight ratio and relatively high pH, salad vegetables host large microbial populations, particularly bacteria, which contribute to the natural decay of vegetative organs detached from the plant (Ragaert et al., 2007). Human pathogens such as *Listeria monocytogenes, Salmonella*, and *Escherichia coli* O157:H7 can contaminate foodstuffs both during plant cultivation and processing (Francis et al., 1999; Brandl, 2006; Franz and van Bruggen, 2008).

Evidence of fresh produce-associated foodborne illnesses has been growing in recent decades and the description of such outbreaks is common in the United States and elsewhere (Berger et al., 2010), despite the prevalence of the main pathogens continuing to be low in fruit and vegetables (EFSA, 2015; Denis et al., 2016). However, outbreaks of illness associated with vegetables and juices have been increasing in recent years, rising from 4.4% in 2013 to 7.1% in 2014 (EFSA, 2015). *Salmonella* spp. and *Listeria* spp. are of major concern, due to the high number of cases and to the severity of the related harm, respectively (EFSA, 2015).

*Salmonella enterica* is an enteric, Gram negative pathogen which can contaminate fresh produce through various routes, including via use of organic waste as fertilizer, contamination of irrigation waters with fecal material, direct contamination by livestock and wild animals and hygiene errors in handling and processing (Heaton and Jones, 2008). *L. monocytogenes* is a ubiquitous Gram positive pathogen frequently isolated from food processing plants and recognized as an important agent of cross-contamination during food handling at both industrial and domestic levels (Lomonaco et al., 2015).

The risk derived from contaminated foodstuffs is increased for YOPIs (Young, Old, Pregnant, Immunocompromised) due to their high susceptibility and, thus, the possibility for disease to be more severe. The increased importance of fresh produce-associated foodborne illnesses has directed the attention of the scientific community toward the implementation of mitigation strategies able to prevent such cases (Chen and Jiang, 2014).

Despite the growing consumption of ready-to-eat vegetables, raw vegetables are still widely consumed in the European Union. This consumption pattern means the measures taken by consumers play a critical role in the prevention of foodborne diseases, as the consumption phase is the last step in the “farm to fork” chain and the only one beyond the official checks performed by the competent authorities involved in assuring food quality. However, according to the scientific literature, consumers are frequently unaware of their role in the prevention of foodborne diseases and underestimate the incidence and severity of such diseases (Losasso et al., 2012). Moreover, it has been shown that improved knowledge allows consumers to make informed choices regarding their actions and, consequently, the accuracy and the extent of information acquired by consumers could be of major significance in foodborne illness mitigation strategies (Losasso et al., 2012, 2013; Faccio et al., 2013).

Among mitigation strategies, sanitation treatments can play an important role in reducing pathogen levels on fresh vegetables. Application of a detergent before disinfection may help remove microbes from the surface of fresh produce (Gil et al., 2009). Most investigations concerning the efficacy of disinfectants for reduction of pathogenic bacteria have been conducted on inoculated fresh fruits (Liao and Sapers, 2000; Bastos et al., 2005; Sy et al., 2005) or vegetables (Hilgren and Salverda, 2000; Hellström et al., 2006). Among these studies, chlorine has proved to be one of the best candidate disinfectants, in association with surfactants.

In response to the current public health concerns associated with the microbiological safety of fresh produce, the aims of the present study were to: (1) assess vegetable sanitation strategies used by consumers, and; (2) determine the efficacy of identified common sanitisers at the consumer and industrial levels for reducing *Salmonella* and *Listeria* in fresh vegetables, taking *Eruca vesicaria* (rocket) as a case study.

## Materials and Methods

### Study Design

The study was divided into two phases. In the first phase, two independent surveys were performed to collect information regarding raw vegetable handling and washing methods in the domestic and industrial environments. Results were used to design the second phase of the study, where real life scenarios for raw vegetable management were evaluated for their effectiveness in reducing *S. enterica* and *L. monocytogenes* contamination.

### Survey of Domestic Raw Vegetable Washing

A questionnaire was administered to a sample of consumers selected on a voluntary basis by snowball sampling. Due to defections that normally occur when people participate on a voluntary basis, not all the subjects answered all the proposed questions. The questionnaire consisted of four questions and is available on request. Questions aimed to collect information about vegetable washing in the domestic environment, paying particular attention to:

– *washing methods* (immersion, running water, other);
– *duration of the washing operation* (less or more than 5 min);
– *number of washing sessions* (one, two, three, or more);
– *chemicals added to the wash water* (vinegar, sodium bicarbonate, common salt, commercial products, none, others).

### Survey of Industrial Raw Vegetable Washing

A survey was conducted to investigate the principal industrial vegetable washing methods by interviewing 20 producers of ready-to-eat vegetables located in the northeast of Italy.

### Washing Protocols

The survey of domestic and industrial methods for leafy green washing and sanitation identified six washing methods that were investigated through an experimental study aimed at assessing their effectiveness in the reduction/elimination of previously inoculated *L. monocytogenes* and *S. enterica*.

The selected washing protocols were:

– washing protocol 1 (WP1): 3 min immersion in an acidic solution (peracetic acid + percitric acid provided by one of the industrial vegetable producers) followed by a 3 min rinse with tap water (chlorine residue <0.2 mg/l as required by National D. lgs 31/01);
– washing protocol 2 (WP2): a single 3 min immersion in tap water;
– washing protocol 3 (WP3): WP2 repeated three times (i.e., in three baths of fresh tap water);
– washing protocol 4 (WP4): 15 min immersion in sodium bicarbonate (2.5%);
– washing protocol 5 (WP5): 3 min immersion in a 5% solution of vinegar (vinegar acidity level 6%) in water;
– washing protocol 6 (WP6): 15 min immersion in a solution of sodium hypochlorite (200 mg/L) followed by a tap water rinse.

### Sample Preparation

Unpacked raw rocket (*E. vesicaria*) was obtained from a local retail store. The rocket used did not contain any detectable *Salmonella* or *Listeria*.

**FIGURE 1:**
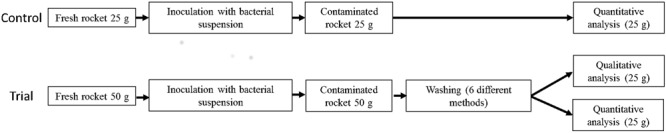
**Flowchart describing the study design of experimental trials**.

Experimental units were composed of 25 g of rocket. For experimental washing trials, two units were pooled before inoculation to have enough material to carry out both quantitative and qualitative microbiological analysis (**Figure 1**).

Two *S. enterica* suspensions were prepared in order to obtain a final microbial load of 3 and 7 Log CFU/g. Suspensions were prepared starting from a mix of three different *S. enterica* isolates belonging to different serovars: *S.* Agbeni (CNRS 463/S03), *S*. Give (4509/2013), and *S*. Derby (4532/2013). The stock cultures were subcultured on tryptone agar slants at 4°C, and *Salmonella* serovars were confirmed by serotyping according to Grimont and Weill (Grimont and Weill, 2007). The *Salmonella* were then transferred to 15 ml of Mueller-Hinton broth (MHB) and incubated at 37°C overnight. Two *L. monocytogenes* suspensions were prepared in order to obtain a final microbial load of 6 and 7 Log CFU/g starting from a mix of one ATCC isolate (ATCC 13932) and two wild isolates from animal matrices. All *Salmonella* and *Listeria* isolates belonged to the collection of pathogenic microorganisms held by the Istituto Zooprofilattico Sperimentale delle Venezie (Legnaro, Italy).

Rocket (25 g) was inoculated with 1 ml of suspension containing *S. enterica* or *L. monocytogenes* and subsequently washed with one of the six washing methods identified by the surveys (see Washing Protocols, above).

### Microbiological Analyses

Microbiological analyses were performed in triplicate according to standard methods. *Salmonella* qualitative analysis was carried out according to ISO 6579:2002/Cor.1 2004 (E) (ISO, 2004b). *Salmonella* quantitative analysis was carried out according to a spread plating method developed in-house: 25 g of rocket were suspended in 225 g of buffered peptone water, and 100 μl of suspension and subsequent dilutions were spread plated on XLD (Xylose-Lysine-Deoxycholate) agar. XLD agar plates were incubated at 37°C for 24 h and then *Salmonella* colonies were counted. The detection limit for this quantitative method was 10 CFU/g. *Listeria* qualitative analysis was carried out according to ISO 11290-2:1998/Amd 1 2004 (ISO, 2004a). *Listeria* quantitative analysis was carried out according to UNI EN ISO 11290-1:2005 (UNI EN ISO, 2005).

### Statistical Analysis

Descriptive analysis was performed in R environment (version 3.2.2). The data were log-transformed and displayed in the form of two scatter plots (Weissgerber et al., 2015) (one for *Salmonella* and one for *Listeria*) containing the outcomes of all examined contamination levels (7 and 3 Log CFU/g for *Salmonella* and 7 and 6 Log CFU/g for *Listeria*). A linear mixed-effects model that allows for inclusion of random effects was used to detect statistically significant differences (significance level of 0.05) among washing methods. In this framework, the within-replicate errors are allowed to be correlated and/or have unequal variances. The analysis was performed using *nlme* package in R environment.

## Results

### Survey of Domestic Raw Vegetable Washing

**FIGURE 2:**
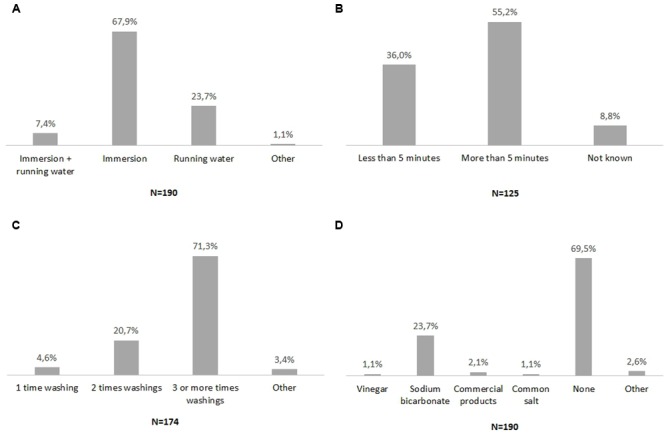
**Survey on domestic vegetable washing. (A)** Vegetable washing methods, **(B)** Duration of vegetable washing, **(C)** Number of washing sessions, **(D)** Chemicals added to water. *N* indicates the number of respondents for each administered question. Percentage of respondents for each possible answer was indicated on the top of the histogram.

Varying numbers of respondents, from 125 to 190 for each question, filled in the four administered questions. The results are reported in **Figure 2**. The most common vegetable sanitation practices were: washing vegetables three times or more; by immersion; for more than 5 min; without any chemicals added to the wash water (**Figures 2A–D**). Moreover, a minor percentage of respondents reported using sodium bicarbonate or vinegar for vegetable sanitation (**Figure 2D**).

Four washing methods proved to be the most commonly used by consumers: immersion of rocket in tap water one time; immersion of rocket in tap water three times; immersion of rocket in a solution of sodium bicarbonate. Interestingly immersion of rocket in a solution of vinegar and water was also used. All of these vegetable washing methods were examined in the second phase of the study.

### Survey of Industrial Raw Vegetable Washing

Altogether, 20 ready-to-eat vegetable producers were interviewed. Two principal methods for washing vegetables were used in all the enrolled production plants. The two methods were: washing vegetables in a solution of sodium hypochlorite followed by rinsing with tap water; washing vegetables in a solution of peracetic and percitric acids, followed by rinsing with tap water. The concentrations of both solutions were not revealed, as they were trade secrets. Both industrial vegetable washing methods were examined in the second phase of the study. The solution of peracetic and percitric acids was provided by one of the producers.

### Effectiveness of Vegetable Washing Methods

**FIGURE 3:**
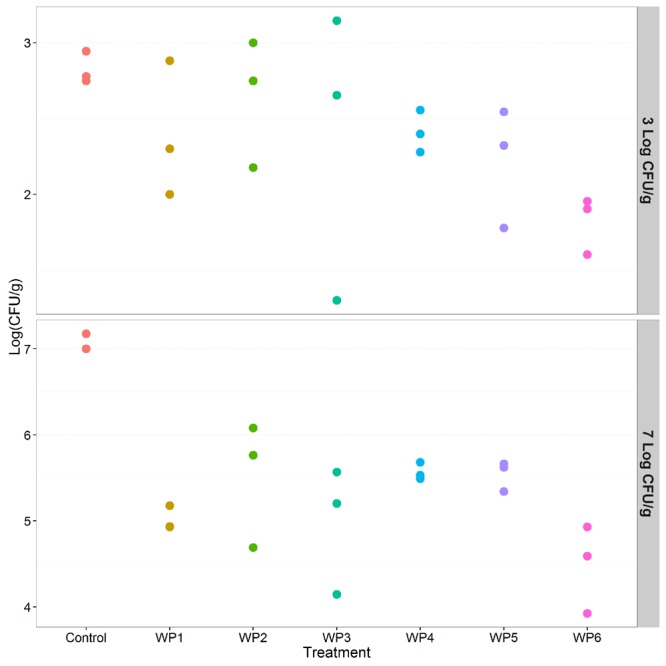
**Effect of the investigated vegetable washing protocols (WP1–WP6) on *S. enterica* counts after artificial contamination on raw *E. vesicaria* (Log scale).** The upper panel shows results obtained in the low contamination condition (3 Log CFU/g); the lower panel shows results obtained in the high contamination condition (7 Log CFU/g).

The 200 mg/L solution of sodium hypochlorite (WP6) resulted in a 2 log reduction of *Salmonella* compared to the control (*p* < 0.001), and in a greater reduction of pathogen counts than all the other washing methods (*p* < 0.001, **Table 1**). However, this effect was observed only in the presence of high *Salmonella* counts (7 Log CFU/g), but it was not observed in the scenario of low level contamination (3 Log CFU/g; **Table 2**). All the other washing methods resulted in non significant reductions of *Salmonella* counts compared to the control, in the presence of both high and low level contamination (**Tables 1** and **2**, **Figure 3**).

**Table 1:** *P* values resulting from the linear mixed-effects model for pairwise comparison of vegetable washing protocols, in relation to the control, for *E. vesicaria* artificially contaminated by *S. enterica* at 10^7^ CFU/g.

	Control	WP1	WP2	WP3	WP4	WP5
WP1	0.799	–	–	–	–	–
WP2	1	0.7332	–	–	–	–
WP3	0.704	1	0.629	–	–	–
WP4	0.748	1	0.676	1	–	
WP5	0.744	0.0562	0.809	0.0350	0.043	–
WP6	<0.001	<0.001	<0.001	<0.001	<0.001	<0.001

**Table 2:** *P* values resulting from the linear mixed-effects model for pairwise comparison of vegetable washing protocols, in relation to the control, for *E. vesicaria* artificially contaminated by *S. enterica* at 10^3^ CFU/g.

	Control	WP1	WP2	WP3	WP4	WP5
WP1	0.987	–	–	–	–	–
WP2	1	0.979	–	–	–	–
WP3	1	0.991	1	–	–	–
WP4	0.998	0.837	0.999	0.996	–	–
WP5	0.553	0.137	0.611	0.517	0.881	–
WP6	0.832	0.998	0.788	0.858	0.480	0.029

**FIGURE 4:**
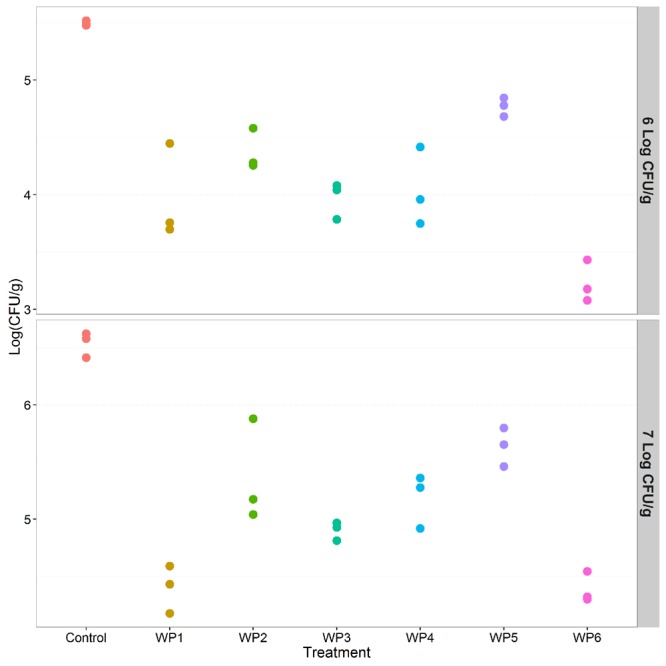
**Effect of the investigated vegetable washing protocols (WP1–WP6) on *L. monocytogenes* counts after artificial contamination on raw *E. vesicaria* (Log scale).** The upper panel shows results obtained in the lower contamination condition (6 Log CFU/g); the lower panel shows results obtained in the higher contamination condition (7 Log CFU/g).

In the case of *Listeria*, results were consistent in the presence of both high and lower contamination levels, 7 and 6 Log CFU/g, respectively. All the washing methods effectively reduced *Listeria* contamination compared to the control (*p* < 0.001) (**Tables 3** and **4**). Sodium hypochlorite solution (WP6) and the solution of peracetic and percitric acids (WP1) produced the greatest *Listeria* reductions: 2 and 1.5 logs, respectively (**Tables 3** and **4**, **Figure 4**).

**Table 3:** *P* values resulting from the linear mixed-effects model for pairwise comparison of vegetable washing protocols, in relation to the control, for *E. vesicaria* artificially contaminated by *L. monocytogenes* at 10^7^ CFU/g.

	Control	WP1	WP2	WP3	WP4	WP5
WP1	<0.001	–	–	–	–	–
WP2	<0.001	<0.001	–	–	–	–
WP3	<0.001	0.001	0.170	–	–	–
WP4	<0.001	<0.001	0.962	0.738	–	–
WP5	<0.001	<0.001	0.772	0.002	0.193	–
WP6	<0.001	1	<0.001	0.086	<0.001	<0.001

**Table 4:** *P* values resulting from the linear mixed-effects model for pairwise comparison of vegetable washing protocols, in relation to the control, for *E. vesicaria* artificially contaminated by *L. monocytogenes* at 10^6^ CFU/g.

	Control	WP1	WP2	WP3	WP4	WP5
WP1	<0.001	–	–	–	–	–
WP2	<0.001	<0.001	–	–	–	–
WP3	<0.001	0.001	0.170	–	–	–
WP4	<0.001	<0.001	0.962	0.738	–	–
WP5	<0.001	<0.001	0.772	0.002	0.193	–
WP6	<0.001	1	<0.001	0.086	<0.001	<0.001

As regards pairwise comparison between treatments, WP1 and WP6 displayed the best performances compared to WP2, WP4, WP5 (*p* < 0.001), and to WP3 (*p* < 0.1). Moreover, WP3 was more effective in reducing *Listeria* counts only compared to WP5.

## Discussion

In this work, the effectiveness of six washing procedures on *S. enterica* or *L. monocytogenes* artificially contaminated onto rocket was assessed. Among the sanitisers investigated, sodium hypochlorite, which has long been used to disinfect fresh produce (Beuchat et al., 1998), produced the largest reductions in microbial counts of both *S. enterica* and *L. monocytogenes*. However, a non-significant effect was obtained from trials involving rocket with low levels of contamination. This was the main limitation of the present study and raises some concerns about washing procedure effectiveness against *Salmonella* in naturally contaminated vegetables.

In the case of *Salmonella*, 200 mg/L sodium hypochlorite significantly reduced bacterial counts, whilst in the case of *Listeria*, all sanitisers significantly reduced the counts, with sodium hypochlorite and combined peracetic and perchloric acids being the most effective. A difference in the effect against *Salmonella* and *Listeria* was observed also in the case of heavy metals such as silver used as antimicrobial (Losasso et al., 2014; Belluco et al., 2016). This could probably be due to the different structure and physiology of *Salmonella* and *Listeria*, being Gram negative and Gram positive, respectively.

Due to its low cost and ready availability, sodium hypochlorite is the most commonly used water disinfectant specifically applied in the fresh-cut produce industry for wash, spray, or flume waters (Gil et al., 2009). However, many disadvantages have been identified when this chemical is used to treat fresh produce. Sodium hypochlorite is highly corrosive and can form carcinogens following reaction with organic matter, particularly at high concentrations (Rodgers et al., 2004). The commonly applied free sodium hypochlorite concentrations in vegetable washing processes range from 50 to 200 mg/L (WHO, 2000). In addition, the use of sodium hypochlorite at high concentrations could generate chlorine gas, which could be harmful for consumers *via* inhalation. Furthermore, the potential for trihalomethane formation by sodium hypochlorite, depending on dose and organic matter content, is well known (WHO, 2000). Evidence on the formation of sodium hypochlorite by-products has been obtained by testing concentrations and/or contact times that would have unacceptable effects on product quality, or could not be applied at industrial scale due to environmental effects and personnel safety issues (WHO, 2000).

The present study demonstrated that the effective concentration of sodium hypochlorite to reduce both *L. monocytogenes* and *S. enterica* contamination on *E. vesicaria* was 200 mg/L, suggesting potential harmful effects for consumers could occur. However, it is difficult to estimate the concentrations of sodium hypochlorite used in real life scenarios by consumers and, consequently, to exclude the risk deriving from consumer exposure to its by-products. Specifically, the amount of sodium hypochlorite added to water is often not measured, but it could exceed the safe level by an order of magnitude.

The current approach to ascertain the safety of foodstuffs is conceptually based on absence (or reduced presence) of foodborne pathogens in processed and unprocessed foodstuffs intended for human consumption, as legally required in the European Union Regulation (EC)2073/2005 (EC, 2005). However, sanitation practices applied to fulfill such safety criteria could change the microbial ecology of food matrices, thus resulting in potential positive and/or negative effects. In fact, due to the complexity of microbial metabolism and the interrelationships between various environmental factors, even apparently insignificant changes in the microbial environment can trigger food safety concerns or drive the evolution of pathogens. For example, the practice of rinsing foods with organic acids, with the aim of eliminating pathogens from the surface, could cause the evolution of acid-tolerant bacterial pathogens better able to survive in a host gastrointestinal tract (Cotter and Hill, 2003). The emergence of microorganisms with combined resistance to biocides and antimicrobial agents is a public health challenge. To date, much evidence has shown that biocides, used indiscriminately in an increasing number of applications, can also play a role in the development (or selection) and dissemination of biocide- and antibiotic-resistant pathogenic bacteria (Dancer, 2014; Tacconelli et al., 2014; Vergnano, 2015).

## Conclusion

Correct vegetable washing practices, both at industrial and consumer levels, are critical to circumvent the phenomena described above. Clearly, this indicates the importance of proper communication campaigns targeting consumers on the correct and safe use of sanitation chemicals during vegetable washing for food safety purposes.

## Author Contributions

APe and SB conceived the experiments and conducted the experiments, CL analysed the results and wrote the manuscript, IP performed data analysis, APi conducted the experiments, DC, RM, and AR conceived the experiments and analysed the results. All authors reviewed the manuscript.

## Conflict of Interest Statement

The authors declare that the research was conducted in the absence of any commercial or financial relationships that could be construed as a potential conflict of interest.

## References

[B1] Bastos M. S. R. de Fátima Ferreira Soares N. José de Andrade N. Cristina Arruda A. Elesbão Alves R. (2005). The effect of the association of sanitizers and surfactant in the microbiota of the Cantaloupe (*Cucumis melo* L.) melon surface. *Food Control* 16 369–373. 10.1016/j.foodcont.2004.04.002

[B2] Belluco S. Losasso C. Rigo L. Conficoni D. Cibin V. Segato S. (2016). Silver nanoparticles as antibacterial towards *Listeria monocytogenes*. *Front. Microbiol* 7:307 10.3389/fmicb.2016.00307 PMC4779933 27014230

[B3] Berger C. N. Sodha S. V. Shaw R. K. Griffin P. M. Pink D. Hand P. (2010). Fresh fruit and vegetables as vehicles for the transmission of human pathogens. *Environ. Microbiol.* 12 2385–2397. 10.1111/j.1462-2920.2010.02297.x 20636374

[B4] Beuchat L. R. Nail B. V. Adler B. B. Clavero M. R. S. (1998). Efficacy of spray application of chlorinated water in killing pathogenic bacteria on raw apples, tomatoes, and Lettuce. *J. Food Prot.* 61 1305–1311. 9798146 10.4315/0362-028x-61.10.1305

[B5] Brandl M. T. (2006). Fitness of human enteric pathogens on plants and implications for food safety. *Annu. Rev. Phytopathol.* 44 367–392. 10.1146/annurev.phyto.44.070505.143359 16704355

[B6] Chen Z. Jiang X. (2014). Microbiological safety of chicken litter or chicken litter-based organic fertilizers: a review. *Agriculture* 4 1–29. 10.3390/agriculture4010001

[B7] Cotter P. D. Hill C. (2003). Surviving the acid test: responses of gram-positive bacteria to low pH. *Microbiol. Mol. Biol. Rev.* 67 429–453. 10.1128/MMBR.67.3.429-453.2003 12966143 PMC193868

[B8] Dancer S. J. (2014). Controlling hospital-acquired infection: focus on the role of the environment and new technologies for decontamination. *Clin. Microbiol. Rev.* 27 665–690. 10.1128/CMR.00020-14 25278571 PMC4187643

[B9] Denis N. Zhang H. Leroux A. Trudel R. Bietlot H. (2016). Prevalence and trends of bacterial contamination in fresh fruits and vegetables sold at retail in Canada. *Food Control* 67 225–234. 10.1016/j.foodcont.2016.02.047

[B10] EC (2005). COMMISSION REGULATION (EC) No 2073/2005 of 15 november 2005 on microbiological criteria for foodstuffs implementing regulation (EC) No 2160/2003 of the European parliament and of the council as regards requirements for the use of specific control methods. *Official J. Eur. Union* L 338 1–26.

[B11] EFSA (2015). The European union summary report on trends and Sources of zoonoses, zoonotic agents and food-borne outbreaks in 2014. *EFSA J.* 13 4329 10.2903/j.efsa.2015.4329 PMC7009540 32625785

[B12] Faccio E. Costa N. Losasso C. Cappa V. Mantovani C. Cibin V. (2013). What programs work to promote health for children? Exploring beliefs on microorganisms and on food safety control behavior in primary schools. *Food Control* 33 320–329. 10.1016/j.foodcont.2013.03.005

[B13] Francis G. A. Thomas C. O’beirne D. (1999). The microbiological safety of minimally processed vegetables. *Int. J. Food Sci. Technol.* 34 1–22. 10.1046/j.1365-2621.1999.00253.x

[B14] Franz E. van Bruggen A. H. C. (2008). Ecology of *E. coli* O157:H7 and *Salmonella enterica* in the primary vegetable production chain. *Crit. Rev. Microbiol.* 34 143–161. 10.1080/10408410802357432 18728991

[B15] Gil M. I. Selma M. V. López-Gálvez F. Allende A. (2009). Fresh-cut product sanitation and wash water disinfection: problems and solutions. *Int. J. Food Microbiol.* 134 37–45. 10.1016/j.ijfoodmicro.2009.05.021 19539390

[B16] Grimont P. A. D. Weill F.-X. (2007). *Antigenic Formulae of the Salmonella Serovars*. Paris: Institut Pasteur.

[B17] Heaton J. C. Jones K. (2008). Microbial contamination of fruit and vegetables and the behaviour of enteropathogens in the phyllosphere: a review. *J. Appl. Microbiol.* 104 613–626. 10.1111/j.1365-2672.2007.03587.x 17927745

[B18] Hellström S. Kervinen R. Lyly M. Ahvenainen-Rantala R. Korkeala H. (2006). Efficacy of disinfectants to reduce *Listeria monocytogenes* on precut iceberg lettuce. *J. Food Prot.* 69 1565–1570. 16865887 10.4315/0362-028x-69.7.1565

[B19] Hilgren J. D. Salverda J. A. (2000). Antimicrobial efficacy of a peroxyacetic/octanoic acid mixture in fresh-cut-vegetable process waters. *J. Food Sci.* 65 1376–1379. 10.1111/j.1365-2621.2000.tb10615.x

[B20] ISO (2004a). *ISO 11290-2:1998/Amd 1 2004–Modification of the Enumeration Medium*. Geneva: ISO.

[B21] ISO (2004b). *ISO 6579:2002/Cor.1 2004 (E) – Microbiology of Food and Animal Feeding Stuffs. Horizontal Method for the Detection of Salmonella spp.* Geneva: ISO.

[B22] Liao C.-H. Sapers G. M. (2000). Attachment and growth of *Salmonella* Chester on apple fruits and in vivo response of attached bacteria to sanitizer treatments. *J. Food Prot.* 63 876–883. 10914653 10.4315/0362-028x-63.7.876

[B23] Lomonaco S. Nucera D. Filipello V. (2015). The evolution and epidemiology of *Listeria monocytogenes* in Europe and the United States. *Infect. Genet. Evol.* 35 172–183. 10.1016/j.meegid.2015.08.008 26254574

[B24] Losasso C. Belluco S. Cibin V. Zavagnin P. Mičetić I. Gallocchio F. (2014). Antibacterial activity of silver nanoparticles: sensitivity of different Salmonella serovars. *Front. Microbiol.* 5:227 10.3389/fmicb.2014.00227 PMC4033309 24904542

[B25] Losasso C. Cappa V. Cibin V. Mantovani C. Costa N. Faccio E. (2013). Food safety and hygiene lessons in the primary school: implications for risk-reduction behaviors. *Foodborne Pathog. Dis.* 11 68–74. 10.1089/fpd.2013.1598 24299005

[B26] Losasso C. Cibin V. Cappa V. Roccato A. Vanzo A. Andrighetto I. (2012). Food safety and nutrition: improving consumer behaviour. *Food Control* 26 252–258. 10.1016/j.foodcont.2012.01.038

[B27] Ragaert P. Devlieghere F. Debevere J. (2007). Role of microbiological and physiological spoilage mechanisms during storage of minimally processed vegetables. *Postharvest Biol.* 44 185–194. 10.1016/j.postharvbio.2007.01.001

[B28] Rodgers S. L. Cash J. N. Siddiq M. Ryser E. T. (2004). A comparison of different chemical sanitizers for inactivating *Escherichia coli* O157:H7 and *Listeria monocytogenes* in solution and on apples, lettuce, strawberries, and cantaloupe. *J. Food Prot.* 67 721–731. 15083724 10.4315/0362-028x-67.4.721

[B29] Sy K. V. McWatters K. H. Beuchat L. R. (2005). Efficacy of gaseous chlorine dioxide as a sanitizer for killing *Salmonella*, yeasts, and molds on blueberries, strawberries, and raspberries. *J. Food Prot.* 68 1165–1175. 15954703 10.4315/0362-028x-68.6.1165

[B30] Tacconelli E. Cataldo M. A. Dancer S. J. De Angelis G. Falcone M. Frank U. (2014). ESCMID guidelines for the management of the infection control measures to reduce transmission of multidrug-resistant gram-negative bacteria in hospitalized patients. *Clin. Microbiol. Infect.* 20 1–55. 10.1111/1469-0691.12427 24329732

[B31] UNI EN ISO (2005). *UNI EN ISO 11290-1:2005 – Microbiology of Food and Animal Feeding Stuffs – Horizontal Method for the Detection and Enumeration of Listeria Monocytogenes – Part 1: Detection Method*. Geneva: ISO.

[B32] Vergnano S. (2015). Decolonization and decontamination. *Curr. Opin. Infect. Dis.* 28 207–214. 10.1097/QCO.0000000000000164 25918955

[B33] Weissgerber T. L. Milic N. M. Winham S. J. Garovic V. D. (2015). Beyond bar and line graphs: time for a new data presentation paradigm. *PLoS Biol.* 13:e1002128 10.1371/journal.pbio.1002128 PMC4406565 25901488

[B34] WHO (2000). *Environmental Health Criteria 216: Disinfectants and Disinfectant By-products*. Geneva: World Health Organization.

[B35] WHO/FAO (2003). Diet, nutrition and the prevention of chronic. *World Health Organ. Tech. Rep. Ser.* 916 i–viii 1–149. 12768890

